# ICTV Virus Taxonomy Profile: *Caliciviridae*

**DOI:** 10.1099/jgv.0.001332

**Published:** 2019-10-01

**Authors:** Jan Vinjé, Mary K. Estes, Pedro Esteves, Kim Y. Green, Kazuhiko Katayama, Nick J. Knowles, Yvan L’Homme, Vito Martella, Harry Vennema, Peter A. White, ICTV Report Consortium

**Affiliations:** ^1^​ Division of Viral Diseases, National Center for Immunization and Respiratory Diseases, Centers for Disease Control and Prevention, Atlanta, GA, USA; ^2^​ Baylor College of Medicine, Houston, TX, USA; ^3^​ CIBIO/InBio – Centro de Investigação em Biodiversidade e Recursos Genéticos, Universidade do Porto, Campus de Vairão, 4485-661, Vairão, Portugal; ^4^​ Caliciviruses Section, Laboratory of Infectious Diseases, National Institute of Allergy and Infectious Diseases, National Institutes of Health, Bethesda, MD, USA; ^5^​ Laboratory of Viral infection I, Kitasato Institute for Life Sciences Graduate School of Infection Control Sciences, Kitasato University, Tokyo, Japan; ^6^​ The Pirbright Institute, Pirbright, Surrey, UK; ^7^​ Université de Montréal, Montréal, Canada; ^8^​ Department of Veterinary Medicine, University Aldo Moro of Bari, Valenzano, Bari, Italy; ^9^​ Centre for Infectious Diseases Control, National Institute for Public Health and the Environment (RIVM), Bilthoven, The Netherlands; ^10^​ School of Biotechnology and Biomolecular Sciences, Faculty of Science, University of New South Wales, Sydney, Australia

**Keywords:** ICTV Report, taxonomy, *Caliciviridae*, norovirus

## Abstract

The family *Caliciviridae* includes viruses with single-stranded, positive-sense RNA genomes of 7.4–8.3 kb. The most clinically important representatives are human noroviruses, which are a leading cause of acute gastroenteritis in humans. Virions are non-enveloped with icosahedral symmetry. Members of seven genera infect mammals (*Lagovirus*, *Norovirus*, *Nebovirus*, *Recovirus*, *Sapovirus*, *Valovirus* and *Vesivirus*), members of two genera infect birds (*Bavovirus* and *Nacovirus*), and members of two genera infect fish (*Minovirus* and *Salovirus*). This is a summary of the International Committee on Taxonomy of Viruses (ICTV) Report on the family *Caliciviridae*, which is available at ictv.global/report/caliciviridae.

## Virion

**Fig. 1.**
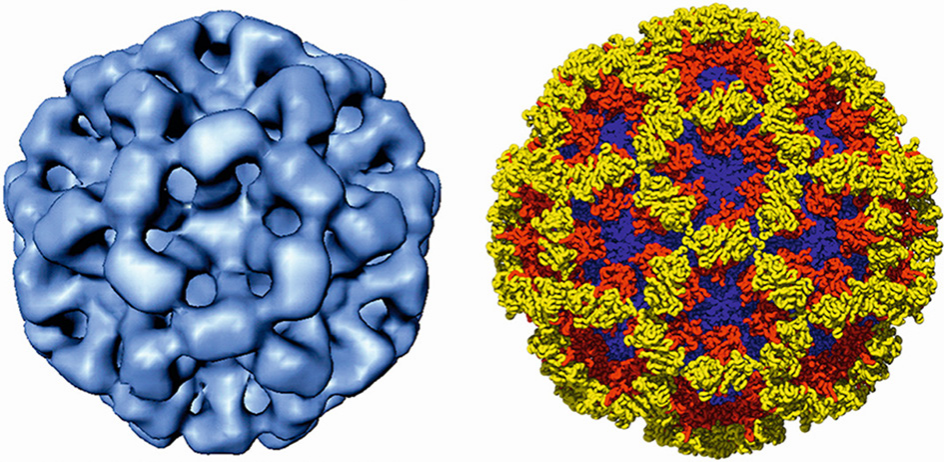
The structure of the calicivirus capsid exemplified by a cryo-image reconstruction of recombinant Norwalk virus-like particles (left). X-ray structure of the Norwalk virus capsid (right) with the shell, protruding 1 and protruding 2 domains shown in blue, red and yellow, respectively. (Courtesy of B. V. Prasad.)

Calicivirus virions are 27–40 nm in diameter, non-enveloped with icosahedral symmetry (Table 1). The capsid is composed of 90 dimers of the major structural protein VP1 arranged on a T=3 icosahedral lattice (Fig. 1) [1]. Caliciviruses are characterised by a capsid architecture with 32 distinct cup-shaped depressions. Generally, caliciviruses are stable in the environment and enteric caliciviruses are acid-stable.

**Table 1.** Characteristics of members of the family *Caliciviridae*

Typical member:	Norwalk virus (M87661), species *Norwalk virus*, genus *Norovirus*
Virion	Non-enveloped with icosahedral symmetry, 27–40 nm in diameter
Genome	Single-stranded, positive-sense genomic RNA of 7.4–8.3 kb, with a 5′-terminal virus protein, genome-linked (VPg) and 3′-terminal poly(A)
Replication	Cytoplasmic
Translation	From genome-sized (non-structural proteins) and 3′-terminal subgenomic (structural proteins) mRNAs
Host range	Mammals (*Lagovirus*, *Norovirus*, *Nebovirus*, *Recovirus*, *Sapovirus, Valovirus* and *Vesivirus*), birds (*Bavovirus*, *Nacovirus*), fish (*Minovirus*, *Salovirus*)
Taxonomy	Realm *Riboviria*; more than ten genera

## Genome

**Fig. 2.**
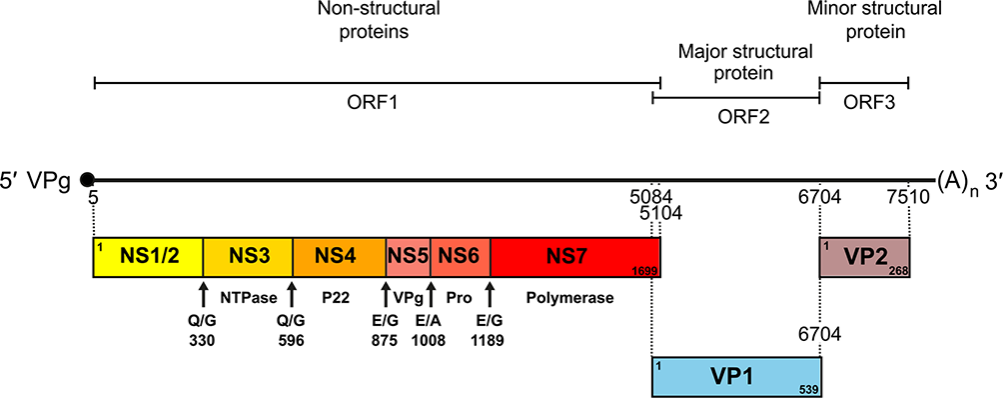
Genome organization of human calicivirus MD-145 (AY032605, 7556 nt, genus *Norovirus*). Protein VPg is covalently linked to the 5′-end of genomic RNA and is depicted by a black circle. Cleavage sites in the ORF1 polyprotein are indicated by arrows; the flanking residues and amino acid coordinates are indicated, although these vary within the family. Pro: protease.

Caliciviruses have a single-stranded, positive-sense genomic RNA of 6.4–8.5 kb organized into either two or three major ORFs, while a further ORF4 of murine norovirus encodes virulence factor 1 (VF1). A protein [virus protein, genome-linked (VPg), 10–15 kDa] is covalently linked to the 5′-terminus of genomic RNAs, which are also polyadenylated at their 3′-termini (Fig. 2). Genus-specific conserved nucleotide motifs are found at the 5′-terminus of ORF1 and at the junction of the coding sequences for the non-structural/structural proteins.

## Replication

Replication of caliciviruses occurs in the cytoplasm in complexes on intracellular membranes by a VPg-mediated translation initiation process unique to the virus family and that uses genomic positive-sense RNA as the template to translate a large polyprotein that undergoes post-translational cleavage by a virus-encoded protease to form at least six mature non-structural proteins (NS1/2, NS3, NS4, NS5, NS6 and NS7) [2]. Subgenomic-sized, positive-sense RNA, co-terminal with the 3′- terminus of the genome, is the template for translation of VP1 as well as the 3′-terminal ORF product VP2 [3]. A dsRNA corresponding in size to full-length genomic RNA has been identified in feline calicivirus, murine norovirus and San Miguel sea lion virus-infected cells, indicating that replication occurs via a negative-sense intermediate. All caliciviruses require VPg; some require the function of eIF4E (feline calicivirus, porcine sapovirus), and some do not (murine norovirus).

## Pathogenicity

Caliciviruses cause species-specific infections, with most noroviruses, sapoviruses and neboviruses restricted to the gastro-intestinal tract; some lagoviruses, saloviruses and vesiviruses cause severe systemic infections in their natural hosts.

## Taxonomy

Viruses of seven genera (*Lagovirus*, *Norovirus*, *Nebovirus*, *Recovirus* [4], *Sapovirus*, *Valovirus* [5] and *Vesivirus*) infect a wide range of mammals, members of two genera infect birds (*Bavovirus* [6] and *Nacovirus* [7]) and members of two genera infect fish (*Minovirus* [8] and *Salovirus* [9]), while caliciviruses have also been detected in the greater green snake and frogs [10], highlighting the wide host range of viruses in the family. Caliciviruses are similar to picornaviruses in the presence of VPg and in sequence similarity of their RNA-directed RNA polymerase and protease proteins.

## Resources

Current ICTV Report on the family *Caliciviridae*: ictv.global/report/caliciviridae.
